# Malassezia species infection of the synovium after total knee arthroplasty surgery

**DOI:** 10.3205/dgkh000279

**Published:** 2016-09-27

**Authors:** Hamed Ebrahimzadeh Leylabadlo, Elham Zeinalzadeh, Najibeh Asl Rahnemaii Akbari, Hossein Samadi Kafil

**Affiliations:** 1 Infectious Disease and Tropical Medicine Research Center, Tabriz University of Medical Sciences, Tabriz, Iran; 2 Department of Parasitology and Mycology, Faculty of Medicine, Tabriz University of Medical Sciences, Tabriz, Iran; 3 Immunology Research Center, Tabriz University of Medical Sciences, Tabriz, Iran; 4 Drug Applied Research Center, Tabriz University of Medical Sciences, Tabriz, Iran

**Keywords:** arthroplasty, infection, knee, Malassezia

## Abstract

Infection is a serious complication after implantation of total knee-prostheses. However, fungal infection is rarely found in periprosthetic joints, and in most reports, the infecting organism is a *Candida* species. This is a case report of infection after left knee total arthroplasty caused by *Malassezia* species. The patient is still undergoing antifungal therapy with voriconazole and is still being followed-up. To the authors’ knowledge, the present case is the first report of *Malassezia* species in a patient after total knee arthroplasty.

## Zusammenfassung

Eine Infektion nach totaler Kniegelenkendoprothesenimplantation ist eine schwerwiegende Komplikation. Pilze sind eine seltene Ursache periprothetischer Gelenkinfektionen und in den meisten Fällen sind Candida spp. die Ursache. Hier wird eine Kasuistik einer Infektion mit *Malassezia* spp. nach totaler Endoprothesenimplantation des linken Knies beschrieben. Der Patient befindet sich noch im Follow-up unter antifungaler Therapie mit Voriconazol. Nach Kenntnis der Autoren ist das der erste Fallbericht einer derartigen *Malassezia*-Infektion.

## Introduction

Reports of fungal infection after total knee arthroplasty (TKA) are extremely rare. In most reports, the infecting organism is a *Candida* spp. [1]. Although yeasts of the genus *Malassezia* (synonym: *Pityrosporum*) are most often associated with tinea versicolor, a superficial disease of the stratum corneum layer of the epidermis, data from several institutions have implicated *Malassezia* as causing a number of more invasive infections in humans, including scalp psoriasis otomycosis and catheter-related fungemia [2], [3]. Subcutaneous mycoses with *Malassezia* are rarer and usually due to dimorphic fungi which are accidentally inoculated into the body after a skin injury or trauma [4]. Here, we report a rare case of *Malassezia* spp. infection of the synovium in a patient after left TKA.

## Case description

**Figure 1:**
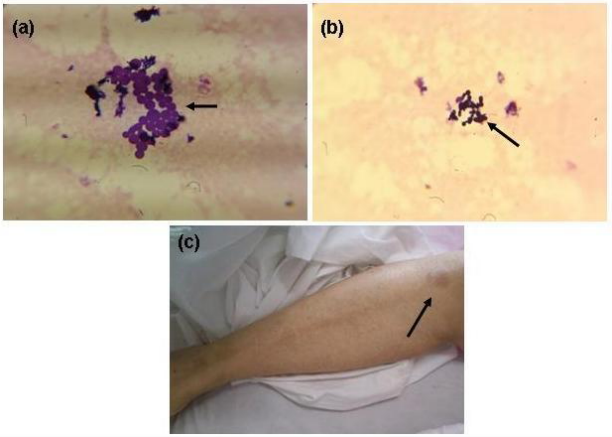
(a), (b) Positive *Malassezia* species from synovial fluid in direct smear with gram staining. (c) Left leg in patient and suspicion of *Malassezia* species in skin.

A 59-year-old Iranian woman presented to the orthopedic department complaining of acute pain, swelling and erythema of her left knee 18 days after undergoing cemented TKA due to left osteoarthritis. Body temperature was 39ºC; blood culture for fungus and bacteria were negative. Laboratory investigations revealed a normal white cell count of 4200 cells/mcl and a C-reactive protein (CRP) of 0.8 mg/L. Synovial fluid collected before antibiotic therapy was mildly cloudy and yellow. Microscopic images of the synovial fluid after Gram-staining revealed some leukocytes and yeast cells with broad pseudohyphae and the morphological appearance of *Malassezia* (Figure 1 (Fig. 1)). The patient was suspected of having had a skin disease on all lower body parts for the past several years. The synovial fluid sample did exhibit bacterial growth on blood agar and MacConkey agar, and it remained negative after 48 h of incubation. The patient started antifungal therapy with amphotericin B (500 mg) and fluconazole (400 mg daily). 

After one dose antifungal therapy (on the third day of hospitalization) with amphotericin B and fluconazole, the CRP changed from 0.8 to 2.33 mg/L. Furthermore, the patient complained of dysuria, with urine cultures revealing *Klebsiella pneumoniae*. The isolates were susceptible to amikacin and cefepime only, and resistant to ceftazidime, ciprofloxacin, gentamicin, imipenem, nitrofurantion, and piperacillin by the disc-diffusion method. Due to urinary tract infection (UTI) and increasing CRP, amphotericin B and fluconazole were discontinued, and cefepime (2 g IV q8hr for 7 days) with voriconazole (200 mg orally every 12 hours) was administered. The UTI improved with the antibiotics. The patient is presently receiving oral voriconazole treatment, and is reporting relief of her symptoms. Her symptoms in the left knee have disappeared; she is still under regular follow-up and in terms of her left knee is presently completely asymptomatic.

## Discussion

Fungal infections reported from 1979 to 2012 after artificial joint replacement include 57 reports with 91 cases; of these, 46 were cases of total knee replacements [5]. Joint infections following arthroplasty are most often bacterial in origin. A deep infection after TKA is a serious complication, but *Malassezia* is an uncommon causative organism in prosthetic joint. *Malassezia* (formerly known as *Pityrosporum*) is naturally found on the skin surfaces of many animals, including humans, and *Malassezia* species inhabit the skin of about 90% of adults without causing harm. Although *Malassezia* are a part of the normal human skin flora, they may also cause or exacerbate several skin diseases, including tinea versicolor, *Pityrosporum* folliculitis, and seborrheic dermatitis [6]. However, *Malassezia* spp. are rarely associated with systemic illness [6]. The existing literature does not contain any evidence of *Malassezia* infection after TKA. 

The patient had no signs or symptoms in the left knee before TKA. The only risk factor for fungal infection was skin disease on lower body parts, which may have constituted a portal of entry for the fungus into the knee joint during surgery. Another possible explanation is the compromised immune system of this patient, but there was no evidence of previously existing underlying disease or malignancy. The patient is presently receiving oral voriconazole treatment, is reporting relief of her symptoms, and a return to normal function of the left knee. She is still being regularly followed-up.

## Conclusion

We report the first case of *Malassezia* infection after TKA, involving the risk factor of *Malassezia* spp. on the patient’s skin. Although prevention strategies such as the administration of prophylactic antibiotics have reduced the incidence of infection after primary arthroplasty, surgeons still encounter this complication frequently. Fungal infections are rare, but this case report illustrates that surgeons should be aware of their possibility and the risk factors involved, and thus exercise peri-operative caution to prevent them.

## Notes

### Competing interests

The authors declare that they have no competing interests.

### Acknowledgements

We thank the staff of Imam Reza Hospital and Qazi Hospital, Tabriz, Iran. All authors were involved in the clinical or pathological and diagnostic management of the patient. All studies of Microbiology Laboratories are supported by Drug Applied Research Center, Tabriz University of Medical Sciences. 

Funding: No specific funding was received from any funding bodies in the public, commercial or nonprofit sectors to carry out the work described in this manuscript.
